# Translation and linguistic validation of 24 PROMIS item banks into French

**DOI:** 10.1007/s11136-024-03690-4

**Published:** 2024-06-12

**Authors:** Sara Ahmed, Emily Parks-Vernizzi, Barbara Perez, Benjamin Arnold, Abigail Boucher, Mushirah Hossenbaccus, Helena Correia, Susan J. Bartlett

**Affiliations:** 1 School of Physical and Occupation Therapy, Faculty of Medicine, McGill University, 3655 Sir William-Osler, Montreal, QC H3G 1Y6 Canada; 2 Centre for Interdisciplinary Research in Rehabilitation of Greater Montreal (CRIR), Montreal, Canada; 3 Lethbridge Layton Mackay Rehabilitation Centre, CIUSSS Centre-Ouest-de-l’Île-de-Montréal, Montreal, Canada; 4 Center for Outcomes Research & Evaluation, Clinical Epidemiology, McGill University Health Center, Montreal, Canada; 5 FACITtrans, Ponte Vedra, FL USA; 6 Department of Medical Social Sciences, Northwestern University Feinberg School of Medicine, Chicago, IL USA

**Keywords:** Patient reported outcomes, Cognitive interviews, PROMIS, Translation, Linguistic validation

## Abstract

**Purpose:**

The Patient-Reported Outcome Measurement Information System (PROMIS®) was developed to provide reliable, valid, and normed item banks to measure health. The item banks provide standardized scores on a common metric allowing for individualized, brief assessment (computerized adaptive tests), short forms (e.g. heart failure specific), or profile assessments (e.g. PROMIS-29). The objective of this study was to translate and linguistically validate 24 PROMIS adult item banks into French and highlight cultural nuances arising during the translation process.

**Methods:**

We used the FACIT translation methodology. Forward translation into French by two native French-speaking translators was followed by reconciliation by a third native French-speaking translator. A native English-speaking translator fluent in French then completed a back translation of the reconciled version from French into English. Three independent reviews by bilingual translators were completed to assess the clarity and consistency of terminology and equivalency across the English source and French translations. Reconciled versions were evaluated in cognitive interviews for conceptual and linguistic equivalence.

**Results:**

Twenty-four adult item banks were translated: 12 mental health, 10 physical health, and two social health. Interview data revealed that 577 items of the 590 items translated required no revisions. Conceptual and linguistic differences were evident for 11 items that required iterations to improve conceptual equivalence and two items were revised to accurately reflect the English source.

**Conclusion:**

French translations of 24 item banks were created for routine clinical use and research. Initial translation supported conceptual equivalence and comprehensibility. Next steps will include validation of the item banks.

## Abstract (de)

There is a rising interest in standardizing the use of patient reported outcomes for Canadians as the importance of measuring patient outcomes as part of value-based and person-centered care increases. This study aimed to translate and linguistically validate 24 PROMIS adult item banks into French that may be used across French-speaking nations. In this work, we outline the processes and draw focus to cultural variations that arise during the language validation and translation procedures. Data from interviews showed that 577 out of the 590 translated items required no revision. Eleven items needed iterations to increase conceptual equivalency. Translating complete PROMIS items banks reveals that while most PROMIS domains are conceptualized and described identically across cultures, a few items required further revisions to establish equivalency. Initial translation supported conceptual equivalence and comprehensibility. French translations for 24 PROMIS item banks are now available for routine clinical use and research. Next steps will include validation of the item banks.

## Introduction

Patient-reported outcome measures offer valuable perspectives on the effectiveness of medical treatments, surgeries, and healthcare services from the perspective of individuals who are directly affected by them, by emphasizing the patient’s perspective. Patient self-reports of health status, including symptoms, quality of life, and day-to-day functioning can support communication and shared decision making during clinical encounters. Such collaborative care is crucial for improving patient engagement, customizing care to each patient’s requirements, and supporting a patient-centred approach to healthcare. PROMs also provide vital information to researchers and healthcare professionals that helps with clinical decision-making, quality improvement, and policy development [1]. Incorporating the patient’s perspective into the evaluation of healthcare using PROMs is needed to improve the value of the care provided relative to the resources and cost needed. In Canada there has been strong support to implement PROMs to guide value-based health care [2].

For nearly 20 years, The National Institutes of Health (NIH) has invested in the development of the Patient Reported Outcomes Measurement System (PROMIS®) [3–6] to advance the measurement, understanding, and use of patient-reported outcomes (PROs) in clinical research and practice. PROMIS tools measure aspects of physical, social, and mental health, in adults and children. It was developed and tested using advanced qualitative and quantitative psychometric approaches across clinical settings. Multiple publications have supported the validity, reliability, and responsiveness of PROMIS measures across different conditions [7–11]. The psychometric properties of PROMIS measures have been shown to be equal to legacy measures in some cases, and more sensitive to symptom change compared to legacy measures [12–14]. More recently, use of PROMIS measures during routine care has been shown to enhance communication and shared decision-making between clinicians and patients [15, 16] and help harmonize the measurement of PROs across countries and research initiatives [17, 18]. The PROMIS Profile measures, which assesses key areas such as pain, fatigue, anxiety, depression, sleep disruption, physical function, and participation in social roles and activities/peer relationships, is available in over 70 languages for adults and 30 for children. Translations for many additional domains are also accessible [19].

PROMIS uses item banks, a set of items that measure the underlying construct, as opposed to traditional measures which present a predefined set of items. A subset of items can be administered as fixed short forms or as a computer adaptive test (CAT). The advantage of CATs is that it adapts to each respondent based on previous responses. The CAT iteratively selects the most suitable items for a respondent to complete based on a predefined level of precision. CATs often yield highly precise and complete information on a given construct with a relatively small number of items thereby reducing respondent burden. (PROMIS item banks and short forms are available at healthmeasures.net.)

Given the increasing importance of measuring PROs as part of value-based and person-centered care, there is a rapidly growing interest to standardize the use of PROs for Canadians. In 2018, we began an initiative to translate and culturally adapt PROMIS item banks to French, starting with adult item banks supported in part by the Canadian Institutes for Health Research’s Strategy for Patient-Oriented Research. Our goal was to increase access to PROMIS measures for researchers and clinicians and as a possible solution to standardize patient-reported outcome measures for national use in clinical care, research, and quality improvement of health services, particularly in bilingual provinces including Quebec, Ontario, and New Brunswick.

The objective of this study was to translate and linguistically validate French versions of 24 PROMIS adult item banks that could be used across French-speaking countries. In this paper, we describe the process and highlight cultural nuances arising during translation and linguistic validation processes.

## Methods

### Promis item banks

The 24 adult PROMIS item banks and scales that underwent English to French translation are listed in Table 1. Domains were prioritized by patients and clinicians from chronic pain management programs and represent the most important domains selected across chronic conditions [20]. All item banks use a 5-point Likert scale and query the past 7 days except for the physical function and participation item banks, which do not use a recall period, the self-efficacy item banks, which ask about the respondent’s current level of confidence and the psychosocial illness impact - positive and negative banks, which ask respondents to consider how their illness has affected them and to rate how true statements are for them before their illness, again now and since their illness.

**Table 1:** The 24 adult PROMIS item banks and scales that underwent English to French translation

Health domain	Item Bank or Scale Name		Number of items translated into French
Mental	1.	PROMIS Bank v1.0 - Emotional Distress – Anxiety	26
	2.	PROMIS - Cancer Bank v1.0 - Anxiety	2
	3.	PROMIS Bank v1.0 - Emotional Distress – Depression	19
	4.	PROMIS - Cancer Bank v1.0 - Depression	7
	5.	PROMIS Bank v1.0 - Psychosocial Illness Impact - Negative	32
	6.	PROMIS Bank v1.0 - Psychosocial Illness Impact – Positive	39
	7.	PROMIS Bank v1.0 - General Self-Efficacy	10
	8.	PROMIS Bank v1.0 - Self-Efficacy for Managing Daily Activities	35
	9.	PROMIS Bank v1.0 - Self-Efficacy for Managing Emotions	25
	10.	PROMIS Bank v1.0 - Self-Efficacy for Managing Medications and Treatments	26
	11.	PROMIS Bank v1.0 - Self-Efficacy for Managing Social Interactions	23
	12.	PROMIS Bank v1.0 - Self-Efficacy for Managing Symptoms	28
Physical	13.	PROMIS Bank v1.0 - Fatigue	65
	14.	PROMIS Bank v1.0 - Pain Behavior	39
	15.	PROMIS Bank v1.1 - Pain Interference	30
	16.	PROMIS - Cancer Bank v1.1 - Pain Interference	3
	17.	PROMIS Scale v2.0 - Neuropathic Pain Quality 5a	5
	18.	PROMIS Scale v2.0 - Nociceptive Pain Quality 5a	5
	19.	PROMIS Bank v2.0 - Physical Function	89
	20.	PROMIS - Cancer Bank v1.1 - Physical Function	6
	21.	PROMIS Bank v1.0 - Sleep Disturbance	15
	22.	PROMIS Bank v1.0 - Sleep-related Impairment	16
Social	23.	PROMIS Bank v2.0 -Ability to Participate in Social Roles and Activities	31
	24.	PROMIS Bank v2.0 - Social Isolation	14
**Total item count**			**590**

*Includes item bank title, instructions, item context, item stem and/or answer scales

### Translation process

The PROMIS Health Organization (PHO) in 2018 provided authorization to the Canada PROMIS National Center (PNC) representatives (SA, SB) to translate 24 adult PROMIS item banks into French. We followed translation recommendations by ISPOR [21] and used best practice methods of FACIT [22, 23] and PROMIS. The translation was conducted in partnership between FACITtrans and the Canadian PNC using the Functional Assessment of Chronic Illness Therapy (FACIT) translation methodology (Table 2) which targets semantic/cultural, content, and conceptual equivalence [22]. Translation methods were based on consensus-based best practices [23, 24]. To meet PROMIS methodological standards, a universal approach to translation was adopted. We aimed to create one French version of item banks that can be used in all French-speaking countries. A benefit of this approach is that the final translations should not require further adaptation for other countries, although additional testing in new countries is recommended.

Interested parties including clinicians and members of the general population were involved in the original development and validation, and during cultural and linguistic validation (through cognitive debriefing). All members of the French translation team met International Organization for Standardization (ISO) 17,100 standards for professional competencies and translator qualifications for linguists and were under contract with FACITtrans. All were native speakers of French with the exception of the Back Translator who was a native speaker of English fluent in French.

**Table 2:** FACIT Translation Process

Steps in Translation Process	Description
1. Forward translations	Two forward translations into French by native speakers from Belgium and Switzerland
2. Reconciliation of forward translations	Reconciliation of the two forward translations by a third native French speaking translator from France
3. Back Translation	One back translation of the reconciled version into English by an English native speaker from Canada.
4. Identify discrepancies between the English source back-translations.	Comparison of source and back-translated versions to identify discrepancies and prepare for subsequent expert review phase
5. Expert review	Three reviews by bilingual experts from Belgium, Canada and France to assess the appropriateness of previous steps.
6. Finalization by language coordinator (LC)	FACITtrans translation manager reviews all translations and highlights issues to be resolved by the expert French LC
7. Harmonization of translations	Harmonization of translations for consistency of meaning across languages. NU MSS participated in the harmonization and final quality review steps of the translation process.
8. Formatting/typesetting of test version in electronic format	FACITtrans prepares translations and places them in electronic format for proofreading by translators and subsequent cognitive interviews
9. Proofreading	Proofreading by two translators, one from Canada and the other from France, working independently from one another and reconciling of proofreading
10. Cognitive debriefing	Cognitive interviews conducted by Canada PNC and data review by FACITtrans with quality review/approval process with NU MSS
11. Certification of the translation	FACIT reviews all final revisions and certified the translations

The translation process was iterative and included native French speakers from Belgium, Canada, France, and Switzerland. Forward translation into French by two native French-speaking translators was followed by reconciliation by a third native French-speaking translator. A native English-speaking translator fluent in French then completed a back translation of the reconciled version from French into English. The source and back-translated versions were then compared by FACITtrans to identify discrepancies and evaluate translation issues, providing guidance to further refine the translations during the subsequent review phase. Three independent reviews by bilingual translators from Belgium, Canada and France were completed to assess the clarity and consistency of terminology and equivalency across the English source and French translations. The translations were then finalized by the lead French Language Coordinator (LC), a native of Canada. FACITtrans conducted a final review of all French translations prior to providing all documentation to the Department of Medical Social Sciences (MSS) at Northwestern University (NU), Feinberg School of Medicine which conducted a quality review of the entire translation process and approved the French translations for linguistic validation. The translations were then formatted into the electronic layout and proofread by two translators, one from Canada and the other from France, working independently from one another. Reconciliation of the proofreading commentary followed resulting in translations which were ready for cognitive interviews during the linguistic validation phase. All phases of the translations’ development from forward translation to finalized translation after cognitive interviews is documented in the French Item Histories housed within FACITtrans’ document management system.

The interview guide was created using FACIT testing administration procedures with a debriefing script to provide interviewers with instructions and cues to administer to participants for the cognitive interviews. Three Canadian research team members from McGill, who were fluent in French, were trained on conducting the interviews and following a standardized process. Eighty members of the general population answered the French version of the paper questionnaire and then were cognitively interviewed using a script developed by FACITtrans and the Department of Medical Social Sciences (MSS) at Northwestern University’s (NU) Feinberg School of Medicine. Participant comments and critiques were used to further refine the translations. Changes made as a result of the interviews were proofread for accuracy. Recruitment and cognitive interviews were carried out by the Canadian PNC at McGill University, located in Montreal, Quebec, the second largest French-speaking city in the world after Paris, France.

Pre-final versions of the French translations were completed by a small sample of French-speaking community participants in Canada (*n* = 80). Respondents country of birth included Algeria (*n* = 1), Bulgaria (*n* = 1), Canada (*n* = 45), France (*n* = 1), Haiti (*n* = 1), India (*n* = 1), Mauritius (*n* = 21), Mexico (*n* = 2), Morrocco (*n* = 3), Romania (*n* = 1), Venezuela (*n* = 1). Two participants did not report their country of birth during the cogntive interview. The participants then took part in individual cognitive interviews conducted in French to assess the relevance, understandability, and cultural appropriateness of the translations.

A qualitative analysis of cognitive interviews for each translated item was used to identify conceptual and linguistic differences between cultures. The synthesis included documenting when during the translation processes the potential issues were found, the frequency with which they occurred, and the manner in which the issue was resolved.

## Results

Twenty-four PROMIS item banks were translated and linguistically validated into French versions. Twenty-eight males and 67 females participated in the cognitive debriefing (Table 3). The average age of participants was 43 years old (range 26–78). Eleven (12%) had a high school level education, and 83 (87%) college/technical degree or higher. Place of birth was mainly Canada (56%), Ile Maurice (14%), and Mauritius (11%).

Of the 590 items translated; 577 items required no revision. The 13 items that were revised spanned multiple domains: Mental (Depression (*n* = 1)), Physical (Fatigue (*n* = 2); Pain Quality – Nociceptive (*n* = 1); Physical Function (*n* = 1)); and Social (Ability to Participate in Social Roles and Activities, (*n* = 6); Psychosocial Illness Impact (*n* = 1); Social Isolation (*n* = 1);). The authors provide a brief description of the items’ revision and the reason for the revision (see Table 4). Eleven of these revised items required iterations to improve conceptual equivalence while two items were revised to reflect the English source accurately.

**Table 3:** Characteristics of cognitive debriefing participants

Characteristic	
Sex *n* (%)	
Female	67
Male	28
**Age Mean (range)**	43 (26–78)
**Place of Birth n (%)**	
Canada	53 (56)
Ile Maurice	13 (14)
Mauritius	10 (11)
Morocco	4 (4)
Venezuela	3 (3)
Mexico	2 (2)
India	1 (1)
Bulgaria	1 (1)
Haiti	1 (1)
France	1 (1)
Algeria	1 (1)
Egypt	1 (1)
Togo	1 (1)
Missing	3 (3)
**Education level n (%)**	
Advanced degree	33 (34)
College degree	22 (23)
Technical degree	28 (30)
High school	11 (12)
Missing	1 (1)

Six items were changed to make the French translation consistent with other banks already translated into French and linguistically validated in previous studies (Table 4). For one of the items, an extraneous concept (“as usual)” was removed from the translation as the concept is not present in the source English. Changes were applied to six of the items for linguistic reasons identified from the cognitive interviews. This included French translations of the words “achy” and “angry” that did not reflect the intended meaning of the English source terms. Two items included idiomatic terms “bushed” and “wiped out” which did not readily translate to French, one item required clarity of the meaning of a “pull-up”, and, finally, a slight revision to French word order was required for the phrase “are around me but not with me.” FACITtrans resolved linguistic and cultural differences by reviewing options with Language Coordinators (LCs) from Canada and France and discussing various French phrasings to convey the intended meaning of the English source. In each case discussion from the translation phase and respondent commentary from the cognitive interviews was referenced during the decision-making process to ensure any issue of miscomprehension was resolved.

**Table 4:** Item revisions

PROMIS domain	PROMIS item bank	No.	English source item	Translation issue	Translation or linguistic validation issue	French test version	Final French version
Mental	Depression v1.0 and Cancer Dep	1.	I felt angry	Linguistic Alternative debriefed, participant preference	Finding the best word in French define “angry”. Consistency maintained with French NQOL translations.	fâché(e)	en colère
Physical	Fatigue v1.0	2.	How bushed were you on average?	Linguistic Alternative debriefed, participant preference	Finding the best word in French define “bushed”.	lessivé(e)	au bout du rouleau physiquement
		3.	How wiped out were you on average?	Linguistic Alternative debriefed, participant preference	“Wiped out” is an idiomatic English expression. Respondents shown alternative and unanimously agreed on alternative	éreinté(e)	anéanti(e)
	Pain Quality - Nociceptive	4.	Did your pain feel achy?	Linguistic	Reflecting the right level of pain intended by the word “achy”	inconfortable	faible
	Physical Function v2.0 and Cancer PF	5.	Are you able to do a pull-up?	Linguistic	Respondents did not understand French translation of “pull-up”. Revision provides a description.	faire une traction	soulever votre corps (faire une traction) en vous agrippant à une
Social	Ability to Participate in Social Roles and Activities	6.	I have trouble doing all of my regular leisure activities with others	Consistency	Made consistent with other banks (I have trouble) and added “personnes” to match English source	J’ai de la difficulté	J’ai du mal
		7.	I have trouble doing all of the family activities that I want to do	Consistency	Made consistent with other banks (I have trouble)	J’ai de la difficulté…	J’ai du mal
		8.	I have trouble keeping up with my family responsibilities	Removal of extraneous concept from translation	The phrase “comme d’habitude” removed from the translation as the concept of “as usual” is not present in the source.	J’ai du mal à assumer mes responsabilités familiales comme d’habitude	J’ai du mal à assumer mes responsabilités familiales
		9.	I have trouble doing all of my usual work (include work at home)	Consistency	Made consistent with other banks (« I have trouble » & “include”)	J’ai de la difficulté…	J’ai du mal
		10.	I have trouble doing all of the activities with friends that I want to do	Consistency	Made consistent with other banks (I have trouble)	J’ai de la difficulté…	J’ai du mal
		11.	I have trouble keeping up with my work responsibilities (include work at home)	Consistency	Made consistent with other banks (I have trouble)	J’ai de la difficulté…	J’ai du mal
	Psychosocial Illness Impact - Positive v1.0	12.	I have a sense of purpose in life	Consistency	Revised to be consistent with a recent NeuroQoL translation of the same item.	but	raison d’être
	Social Isolation v2.0	13.	I feel that people are around me but not with me	Linguistic	Respondents did not understand the original translation. Small adjustment made to clarify the source meaning.	sont autour de moi, mais pas avec moi	autour de moi ne sont pas avec moi

## Discussion

We translated and linguistically validated 24 PROMIS item banks with 590 items into a French version validated by native French speakers in Quebec, Canada. A total of 13 items (2%) required revisions to ensure conceptual and cultural equivalence. The translations followed internationally recognized guidelines and extensively involved interested parties in the translation and cultural and linguistic validation process.

There are some vocabulary differences between the French language spoken in Canada and other French-speaking countries. There are specific Canadian French terms that differ from France such as “depanneur” (used in Canada) and “épicerie” (used in France) which are distinct terms used in each respective country to mean “convenience store.” In the PROMIS Physical Function item bank, the term “pull-up” required careful consideration of terminology when developing the French translation. During the translation phase, the technical French term “faire une traction” (to do a pull-up) was approved by the French translation team, but there was concern from the Canadian LC that members of the French-speaking community in Canada might have difficulty understanding. During the cognitive interviews Canadian respondents indicated they did not, in fact, understand the term as intended by the English source. A revision using the technical term paired with a description of what a “pull-up” means was implemented to ensure universal comprehension (soulever votre corps (faire une traction) en vous agrippant à une barre fixe” (to lift your body up (do a pull-up) while gripping a fixed bar). The inclusion of translators and reviewers from Canada and France was essential to ensuring a French version was developed. When using a universal translation approach, focusing on the similarities of the language used in the various regions, rather than differences helps to avoid miscomprehension. Additionally, further linguistic validation is recommended in other French-speaking regions to confirm linguistic, cultural, and conceptual equivalence of the translations.

Idioms that are culturally acceptable in English-speaking regions needed to be replaced with French translations that could be understood in French-speaking countries while remaining conceptually equivalent to the English source. Words such as “bushed” and “wiped out” could not be translated literally and equivalent expressions “au bout du rouleau physiquement” (physically at the end of one’s rope) and “anéanti(e)” (wiped out) respectively were needed for the French translations.

We have described the iterative steps employed in the rigorous methodology (multiple forward and backward translation, reconciliation, independent review, finalization, proofreading) and cross-cultural validation process to translate multiple English PROMIS item banks into French for use in French-speaking countries. We engaged native speakers in both English and French in Belgium, Canada, France, and Switzerland, and utilized both content experts and members of the general population to ensure the development of culturally relevant items. After obtaining authorization to conduct the translations, we worked closely with FACITtrans and the Department of Medical Social Sciences (MSS) at Northwestern University (NU) Feinberg School of Medicine, to help guide the process and ensure best practices were followed to ensure equivalence of meaning and measurement between English and French versions, and among French-speaking countries. Most items were easily translated and culturally relevant with only a minority of items, most addressing aspects of social function, requiring additional consideration or adaptation.

Finally, the translation process involved choices akin to those in other language translations concerning measurements of distance, time, and idiomatic expressions. Measurements frequently require context and qualitative descriptions to be clear [25–27]. A rigorous translation approach ensures that subtle nuances between languages and idiomatic expressions within countries that speak the same language are addressed to ensure that the items in PROMIS item banks are consistent, comparable, and perform similarly across different languages. Future steps will include differential item functioning (DIF) analyses by language, and further in-depth psychometric analyses in French-speaking countries.

## Abbreviations

PROMIS®: Patient Reported Outcomes Measurement System
PRO: Patient-reported outcome
CAT: Computer adaptive test
PHO: PROMIS Health Organization
PNC: PROMIS National Center
FACIT: Functional Assessment of Chronic Illness Therapy
ISO: International Organization for Standardization
MSS: Department of Medical Social Sciences
NU: Northwestern University
LC: Language Coordinator
DIF: Differential item functioning
